# Paying the costs of reproduction

**DOI:** 10.7554/eLife.09556

**Published:** 2015-07-28

**Authors:** Thomas Flatt

**Affiliations:** Department of Ecology and Evolution, University of Lausanne, Lausanne, Switzerlandthomas.flatt@unil.ch

**Keywords:** intestine, organ plasticity, stem cells, *D. melanogaster*

## Abstract

When a female fly mates it produces a hormone that increases the size of its midgut and enhances fat metabolism in order to provide the energy needed for reproduction.

**Related research article** Reiff T, Jacobson J, Cognigni P, Antonello Z, Ballesta E, Tan KJ, Yew JY, Dominguez M, Miguel-Aliaga I. 2015. Endocrine remodelling of the adult intestine sustains reproduction in *Drosophila*. *eLife* **4**:e06930. doi: 10.7554/eLife.06930

**Image** The ovaries, midgut and fat body–which all store lipids–are located close to each other in the fly's abdomen

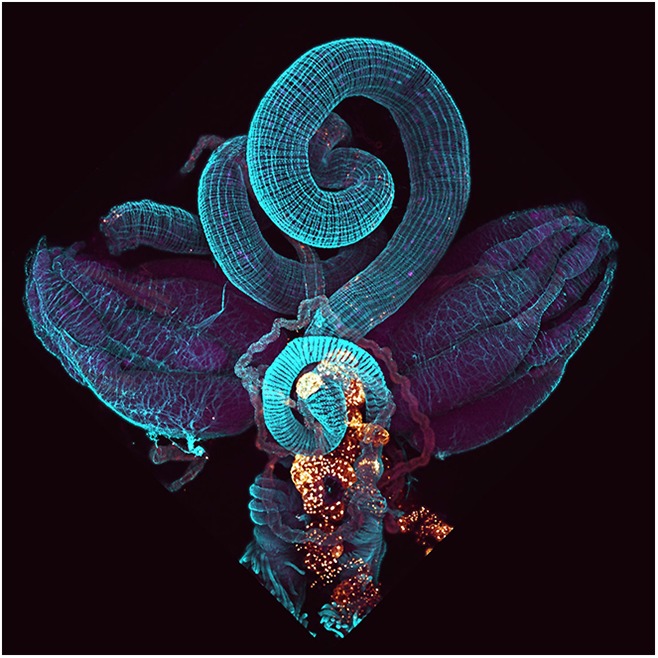

It has been known for a long time that reproduction has profound effects on animal physiology. Around 335 BC, Aristotle noted that many animals grow larger and live longer if they have been castrated, which suggests that reproduction can reduce growth and survival (Leroi, 2014). George C Williams formalized this concept in 1966, arguing that organisms are subject to ‘trade-offs’ between the energetic demands of reproduction and the energy required to survive (Williams, 1966).

Many animals consume much of their fat reserves during reproduction, and molecules of fat—known as lipids—are a major ‘currency’ through which the energy costs of reproduction are paid (Schneider, 2004; Hansen et al., 2013). However, the molecular mechanisms whereby reproduction affects metabolism remain poorly understood. Now, in *eLife*, Irene Miguel-Aliaga at Imperial College London, Maria Dominguez at Universidad Miguel Hernández and co-workers—including Tobias Reiff as first author—have used the fruit fly *Drosophila* as a model to gain insights into the physiology of reproductive investment after mating (Reiff et al., 2015).

Reiff et al. report that mating triggers many changes in the midgut of female flies that enhance reproductive success. The midgut—which has a similar role to the small intestine of mammals—increases in size due to the division of ‘progenitor’ cells. Also, genes involved in the metabolism of lipids are up-regulated in enterocytes, which are the cells responsible for absorbing nutrients in the midgut. This remodeling of the midgut mirrors findings from mammals where internal organs such as the gastrointestinal tract increase in size during reproduction, perhaps to enhance the uptake of nutrients (Speakman, 2008). But what are the molecular mechanisms that underlie these profound changes in the fly?

A good candidate is a molecule contained in male semen called sex peptide. Upon mating, sex peptide leads to increases in feeding and egg production in the female fly, but also suppresses immune responses and reduces lifespan (Kubli and Bopp, 2012; Short et al., 2012). Several of these changes seem to occur because sex peptide increases the production of a hormone called juvenile hormone (Flatt et al., 2005; Yamamoto et al., 2013).

Reiff et al. significantly extend this model by confirming that mating increases the levels of juvenile hormone, and showing that this hormone is responsible for the observed changes in female flies after mating (Figure 1). Treating unmated females with a molecule that mimics juvenile hormone increases the size of the gut and alters the metabolism of lipids: however, these changes do not happen if the gland that produces juvenile hormone is removed.

**Figure 1.**
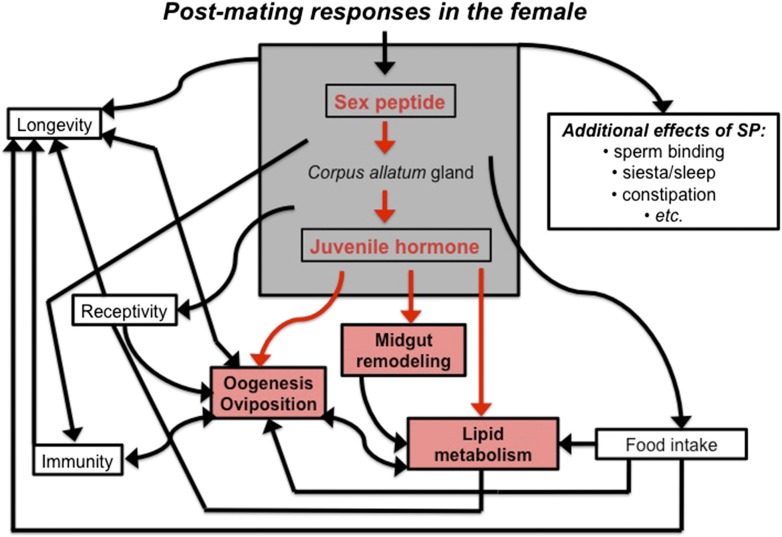
The sex peptide-juvenile hormone model of mating. Sex peptide (SP) and juvenile hormone alter food intake, receptivity to mating, egg production (oogenesis), immunity, lifespan and many other aspects of female physiology upon mating (shown in black). There is evidence that both sex peptide and juvenile hormone can influence many of these processes. Although sex peptide induces the production and release of juvenile hormone, it is not clear whether all the effects of sex peptide on the females are due to the release of this hormone. Reiff et al. add to this model by showing (highlighted in red) that remodeling of the midgut, increased lipid metabolism, egg production and other important changes in female flies depend upon juvenile hormone signaling, which increases after mating (Reiff et al., 2015).

Next, Reiff et al. test whether two receptors that bind juvenile hormone are required for gut remodeling. By silencing the genes for these receptors (called *Met* and *gce*) in the midgut using a technique called RNA interference, Reiff et al. find that juvenile hormone signals directly to the cells of the midgut. This, in turn, stimulates the progenitor cells to divide and increases lipid metabolism in enterocytes. But how does the remodeling of the midgut impact on reproduction?

Again, juvenile hormone seems to be instrumental. It is involved in various aspects of egg production, but no role for juvenile hormone in the gut during reproduction has previously been reported. Reiff et al. now show that down-regulation of *gce* in enterocytes reduces egg production; this indicates that the gut remodeling triggered by juvenile hormone increases reproductive success.

Animals that have had their ovaries removed increase the amount of fat they store after mating (Hansen et al., 2013). Similarly, Reiff et al. show that female flies that have been genetically sterilized increase the size of their midgut and accumulate more fat after mating than virgin females. Remarkably, the same effect is observed when unmated sterile females are treated with a compound that mimics juvenile hormone. Thus, even when reproduction is abolished, juvenile hormone is sufficient to increase midgut size and fat storage.

The advances made by Reiff et al. prompt a number of fascinating questions. Are the changes seen in the female flies all due to sex peptide? Is altering lipid metabolism the only way that juvenile hormone enhances reproductive success? Does juvenile hormone signaling in the midgut affect immune responses and other juvenile hormone-dependent processes? Recent evidence suggests that juvenile hormone can alter gene expression and lifespan independently of its effects on reproduction (Yamamoto et al., 2013). Therefore, is it possible to separate the negative effects of juvenile hormone on the body from the benefits to reproduction? And, does the degree of gut remodeling in response to mating vary between flies and populations of flies, or is it always the same?

Whatever future work reveals, the current study clearly shows that mating triggers a highly complex cascade of effects that precede egg production. These changes are regulated by juvenile hormone and prepare the female fly's body for the energy demands of reproduction. More generally, the results of Reiff et al. support the idea that hormones are key regulators of the energy invested into reproduction.
